# What contributes to medical debt? Evidence from patients in rural China

**DOI:** 10.1186/s12913-020-05551-5

**Published:** 2020-07-28

**Authors:** Yanjiao Xin, Junnan Jiang, Shanquan Chen, Fangxu Gong, Li Xiang

**Affiliations:** 1 School of Medicine and Health Management, Huazhong University of Science and Technology, 13 Hangkong Road, Qiaokou District, Wuhan, 430030 China; 2 School of Clinical Medicine, University of Cambridge, Cambridgeshire, UK

**Keywords:** Rural China, Medical debt, Critical illness insurance, Social support, Two-part model

## Abstract

**Background:**

Rural households in developing countries usually have severe medical debt due to high out-of-pocket (OOP) payments, which contributes to bankruptcy. China implemented the critical illness insurance (CII) in 2012 to decrease patients’ medical expenditure. This paper aimed to explore the medical debt of rural Chinese patients and its influencing factors.

**Methods:**

A questionnaire survey of health expenditures and medical debt was conducted in two counties of Central and Western China in 2017. Patients who received CII were used as the sample on the basis of multi-stage stratified cluster sampling. Descriptive statistics and multivariate analysis of variance were used in all data. A two-part model was used to evaluate the occurrence and extent of medical debt.

**Results:**

A total of 826 rural patients with CII were surveyed. The percentages of patients incurring medical debt exceeded 50% and the median debt load was 20,000 Chinese yuan (CNY, 650 CNY = US$100). Financial assistance from kin (*P* < 0.001) decreased the likelihood of medical debt. High inpatient expenses (IEs, *P* < 0.01), CII reimbursement ratio (*P* < 0.001), and non-direct medical costs (*P* < 0.001) resulted in increased medical debt load.

**Conclusions:**

Medical debt is still one of the biggest problems in rural China. High IEs, CII reimbursement ratio, municipal or high-level hospitals were the risk determinants of medical debt load. Financial assistance from kin and household income were the protective factors. Increasing service capability of hospitals in counties could leave more patiemts in county-level and township hospitals. Improving CII with increased reimbursement rate may also be issues of concern.

## Background

In 2010, the World Health Organization pointed out that one of the fundamental functions of a health system is to protect the population against financial risks associated with ill health and countries are obliged to reduce the financial burden on individuals in obtaining necessary health services to achieve universal health coverage (UHC) [1]. However, in most low- and middle-income countries, these conditions are not met, and effective and affordable health services are not accessible to the entire population [2].

High out-of-pocket (OOP) health payment is the most important challenge hindering the achievement of UHC [3, 4]. Studies have indicated that OOP payments constitute a major financial barrier to health utilization, especially for rural households who are poorly equipped to deal with the financial consequences resulting from catastrophic illnesses compared with urban households [5–7]. The health burden of patients in rural China has always been a concern.

From 1950s to 1970s, the rural cooperative medical system (CMS) was the main insurance scheme in rural China. It was primarily financed by the welfare fund of the communes to pay village doctors to deliver primary care and provide prescription drugs. Until mid-1970, 90% of farmers were covered by the CMS [8]. However, this system collapsed in 1979 due to social reform [8]. Since then, Chinese farmers have no medical insurance, and all their medical expenses are paid OOP. In 2003, 96% of rural households in China lacked medical insurance, 38% of the sick did not seek medical attention, and medical debt forced many households to reduce food consumption [9]. The ability to pay became an important determinant of access to healthcare. Large negative health shocks reduced the annual income in rural China by approximately 12.4% [10].

China’s social health insurance scheme currently has three types: the rural new cooperative medical scheme (NCMS, covers rural residents), urban resident-based basic medical insurance (URBMI; covers the urban non-employed, such as students, children, and the elderly), and urban employee-based basic medical insurance (covers urban employees). The enrollment rate of NCMS gradually increased and achieved 98.8% in 2015. In 2016, the government combined NCMS and URBMI into one medical insurance for urban and rural residents. However, the financial protection from NCMS is still insufficient due to limited coverage for outpatient services, weak control of sharply increasing costs, and relatively low benefit levels [11, 12]. The economic burden induced by OOP remains high, and 44.1% of underprivileged households are impoverished because of illness [13]. For the service delivery system, a three-tier public provision system composed of village clinics, township health centers (THCs), and county hospitals was organized in rural China. Provincial and central hospitals provide high-level referral care [14].

In 2012, China implemented critical illness insurance (CII) as a supplementary of basic medical insurance to reduce the OOP payments of patients. CII provides a single lumpsum payment to patients diagnosed with severe and/or critical diseases [15, 16]. It is well developed in many countries, such as the UK and the US [17]. In rural China, CII is a specific reimbursement scheme for patients with high medical expenses. CII was funded by 5–10% of the total basic medical insurance funds or 10–35 Chinese yuan (CNY) per beneficiary in 2015. CII provides an additional 50–70% reimbursement rate for expenses not covered by basic medical insurance. Commercial insurance companies are involved in CII and play an important role in monitoring and supervising health service provisions [18].

One of the problems caused by high OOP expenses is medical debt. Rural households in developing countries always have limited savings to pay for healthcare, and most of them depend on borrowing money to pay for OOP health expenses [19–21]. For example, in a rural area of Cambodia, 62% of families with initial debts borrowed money to pay for treatment and continued to pay high interest rates (ranging between 2.5 and 15% per month) 1 year later; several households even had to sell their land to pay their debts [22]. Krishna reported that health and health-related expenses accompanied by high-interest debt are the largest factor associated with income decline and poverty [23]. Health-related expenses and indebtedness due to OOP payments are major factors responsible for poverty and main barriers to necessary healthcare [24–27]. Medical debt may also decrease a person’s ability to secure loans by negatively affecting his credit score [24]. Research showed that medical debt is one of the main causes of bankruptcy [28].

The medical expenses of urban and rural residents in China are constantly increasing. The statistical yearbook shows that the per capita medical expenses increased from 361 CNY (56 USD) in 2000 to 3784 CNY (582 USD) in 2017, an increase of more than 10 times, while the consumer price index in 2017 was 2.644 times higher than that in 2000 [29]. With the changes in health insurance, the percentage of total health expenditure paid OOP in China increased from 20% in 1978 to 60% in 2001, dropped to 40% in 2008 [30], and then decreased to 28.77% in 2017. A comprehensive assessment of disease burden in China between 1990 and 2010 showed that the leading causes of disability-adjusted life years in 2010 are cardiovascular diseases, cancers, and critical illnesses that lead to high medical expenses [31].

Poverty occurs when the household income is lower than the poverty line, after excluding household consumption expenditures (including OOP expenses). When families are unable to pay for their medical expenses, they choose to borrow money and generate medical debt, which increases household poverty and places a long-term burden on families. CII provides additional compensation to patients who incur high medical costs. Residents not covered by CII may face medical debt, while those covered by CII may not necessarily generate medical debt.

Impoverishment studies, especially on catastrophic health expenditure, have been widely available in China in recent years. Several studies proved that poverty due to illness is very serious in China and health insurance and payment method are the determinants of catastrophic health expenditure [4, 32, 33]. Although medical debt is very serious in China, relevant research is scarce. Only few studies mentioned that high medical OOP costs induce medical debt, but the specific proportion and amount of debt are not mentioned [32, 34].

The distribution of medical debt and its influencing factors, which reflect the mechanisms on how health payments induce poverty, must be understood to shed light on the level of financial protection that a healthcare financing system provides. Moreover, most studies on health-related debt were based on all types of patients [25, 35]. Whether patients with CII have similar influencing factors remains unknown. With the high prevalence rate of critical diseases, such as cancer and cardiovascular diseases, more and more people face high medical expenses that could aggravate their and their family’s economic burden and eventually lead to bankruptcy [36, 37]. The present paper aimed to explore the situation of medical debt among patients in two representative rural areas in Central and Western China and explore the factors related to this situation.

## Methods

### Setting

According to a study by Meng Q et al. [5], households in Central and Western China are more likely to suffer from high rates of catastrophic medical expenditures than those in other regions. Therefore, the present study focused on rural patients from these two regions. Hubei Province in the Central region and Guizhou Province in the Western region were selected as the sample provinces due to the availability of data. In these two provinces, two sample counties were randomly selected: Xiantao (XT) and Yuqing (YQ). XT, where the study sample was drawn, is a county in Southwest Hubei. It had a GDP per capita of 62,601 CNY (nearly 9632 USD) in 2017 and ranks 59th among the top 100 counties (cities) with the largest gross domestic product (GDP) in China. YQ is located in Western China; it has poor resources and a GDP per capita of 31,503 CNY (4744 USD) in 2017.

Both sample cities customized their own CII project under local contexts. In XT, the official CII premium is 12,000 CNY, and the reimbursement rates are 55, 65, and 70% for OOPs of 12,000–30,000, 30,000–100,000, and 70% over 100,000 CNY, respectively. Given the difference in local economic conditions, the deductible in YQ is 8000 CNY, and reimbursement rates are 50 and 60% for OOPs of 8000–60,000 and over 60,000 CNY, respectively. In 2016, the populations of XT and YQ were 1,563,500 and 305,000, respectively, and their inpatient populations were 100,288 and 35,189, respectively. The number of CII beneficiaries in XT was 4137, while YQ had 935.

### Data collection

Data were obtained from a cross-sectional survey carried out in July 2017. The NCMS database from January 2016 to December 2016 for both counties and the collected medical costs and addresses of all patients were obtained. The patients were sampled on the basis of multi-stage stratified clustering and probability proportionate to size (PPS) sampling. In each sample county, five townships were randomly chosen, and the sample size of each township was determined in accordance with PPS sampling. A total of 1000 patients were surveyed in the two regions, with 500 each. The inclusion criterion required that patients benefited from CII in 2016, that is, the OOP payments after NCMS exceeded the CII deductible. The NCMS hospitalization compensation database and the medical assistance database (formerly managed by the Ministry of Civil Affairs) in the sample areas were collected. The patient’s name and ID card were used as the unique identification code to obtain details of the patient’s medical assistance.

Survey was performed using a structured questionnaire, which was constructed in reference to the National Health Services Survey Questionnaire [38] and China Health and Retirement Longitudinal Study questionnaire [39]. The questionnaire used in this study consisted of four parts: patient demographics, inpatient services and expenses, outpatient services and expenses, and medical debts. Data were collected through face-to-face interviews conducted by qualified investigators who received rigorous training before the survey. The investigators were assisted by local health administrators, who were knowledgeable of the whole investigation and only responsible for explaining the purpose of this survey, to dispel the doubts of the respondents and ensure data reliability. Thus, the possibility of bias was minimal. The purpose of the survey were explained to the respondents, who gave their consent to participate in the investigation. Quality control was implemented by the supervisors in charge of guiding and inspecting every step of the survey. The questionnaire developed for this study is provided as Additional file 1.

### Definitions

#### Medical debt

Medical debt is defined as personal debt derived from healthcare expenditures or paying for medical OOP expenses by borrowing money; thus, medical debt was interchanged with borrowing money in this study [22]. The outcome of medical debt was based on the participant’s response to the following question: Have you or has anyone in your family had to borrow money or go into debt because of your critical illness, its treatment, or the long-term effects of that treatment?

#### Coping strategy

According to previous studies, the coping strategies for medical expense were income, savings, borrowing, and asking help from family members [20, 40]. In the present paper, the means the household employed to pay for OOP expenses over the previous year were asked. Such means included the following: (i) savings (including income); (ii) borrowing; (iii) financial assistance from kin; and (iv) others.

### Variables

Previous studies have explored the relationship between various variables and medical debt. For example, Thomas P. O’Toole analyzed the correlation between medical debt and 14 variables, including gender, age, and income [12]. Seifert found that females, the elderly, and middle-class people are more likely to incur medical debt. Christy revealed that individuals with post-secondary education or non-homeowners are more at risk of medical debt [24].

On the basis of previous studies and expertise, four demographic variables were collected (Table 1). Region, gender, and marriage were the dichotomous variables. Age was the continuous variable and divided into four categories. Details on household size, which was defined as the number of people living in the household and sharing functions of production, consumption, and reproduction, were also collected [41]. In accordance with the *Report on the family development in China* [42], the patients were divided into two groups: ≤ 3 and > 4. Per capita household income (PCHI), which is widely used in the literature, was divided into four levels, with the lowest quintile corresponding to level 1 [25, 43]. Whether patients go to hospitals out of the county was a concern due to the severity of CII. Thus, hospital level was divided into two types: county or low level and municipal or high level. Hospital level was a proxy variable that reflected the quality of healthcare patients pursued. NCMS and CII reimbursement were the compensation paid by the NCMS and CII, respectively. NCMS and CII reimbursement ratio was the percentage of reimbursement over inpatient expenses. OOP is the medical payments that patients need to pay by themselves. In our study, it comprised the expenses below the deductible, the expenses above the deductible co-paid by patients, and the non-reimbursable amount beyond the NCMS benefit packages. Other dichotomous variables, such as medical assistance and financial assistance from kin, were also analyzed and simply measured as yes or no.

**Table 1:** Independent variables descriptions

Variables	Description
Region	XT and YQ
Gender	Male = 1, female = 2
Age (year)	The age was divided in 3 groups: < 18, 18–44,45–64,≥65
Marriage	Marriage status was divided into married and others (contains unmarried, divorced and widowed)
Household size	Household size was divided in two groups:≤3 and > 4
Per capita household income	PCHI was divided into four quartiles, level 1 to level 4.
Hospital level	County or less level, municipal or higher level
Inpatient times	The times of inpatient services in past years, continuous variable
Inpatient days	The days of inpatient services in past years, continuous variable
Inpatient expenses	The total expense during hospitalization, continuous variable
NCMS reimbursable expenses	NCMS reimbursement, continuous variable
CII reimbursable expenses	CII reimbursement, continuous variable
Non-direct medical costs (NDMC)	Included transportation costs, board and lodging and other costs relating to healthcare but not included in health expenditures
Indirect costs	The costs associated with time lost by the patients and their family members
Out-of-pocket payments	Continuous variable
Medical assistance	Received medical assistance = 1, other = 2
Financial assistance from kin	Received financial assistance from kin = 1, other = 2

### Data analysis

Data observations had a cluster at zero. First, when incurring health expenses, patients determine whether to pay for OOP expenses by borrowing money, which is a dichotomous event. Second, a large difference in medical debt load exists in patients with medical debt, which is a continuous non-normal distribution variable. Several zeroes in medical debt destroy the normality assumption of random error [44]. Duan et al. [45] proposed two models to solve this problem: the logit model, which estimates whether a person has had medical expenses, and the linear model, which estimates the medical expenses in non-zero coefficients. In the present study, a two-part model was used to explore the factors related to the high likelihood of incurring medical debt. In the two-part model, the first part was a logit regression model and the second part was a logarithm linear regression model because medical debt load satisfies the lognormal distribution. Some independent variables in the regression model were deleted using correlation test. Random effect analysis on counties was included in the regression model. It considered the correlated nature of samples measured from the same county.

## Results

### Demographics of respondents

As shown in Table 2, in 2016, most patients with critical illness aged 44–64 years (51.3% in XT and 42.3% in YQ). The mean numbers of household size in XT and YQ were 3.60 and 4.06, respectively. More patients sought medical care at municipal or high-level hospitals. The median IE in the two counties was 56,159 CNY (8640 USD), considerably higher than PCHI at only 6250 CNY (962 USD). The average NCMS reimbursement rate was 34,742 CNY (5345 USD). The patients in XT had high CII reimbursement rate (8045 CNY or 1238 USD) than those in YQ (5908 CNY or 909 USD), and the difference was statistically significant (*P* < 0.001). The average NDMC of the two counties was 4433 CNY (682 USD), and their average indirect cost was 9244 CNY (1422 USD). The average OOPs in XT and YQ were 32,531 CNY (5005 USD, median = 25,050 CNY or 3854 USD) and 24,100 CNY (3708 USD, median = 18,225 CNY or 2804 USD), and the difference was statistically significant (*P* < 0.001). The percentages of patients acquiring financial assistance from kin were 33.9% in XT and 22.0% in YQ, and the medical assistance were 32.5 and 14.7%, respectively. The percentages of patients with critical illness incurring medical debt exceeded 50%, and the median debt load was 20,000 CNY (3077 USD) in both counties (Table 2).

**Table 2:** Characteristics of total respondents in 2016

Variables	category	Total	XT(*n* = 431)	YQ(*n* = 395)	t /Chi-square tests
		Number (%)	Number (%)	Number (%)	
Gender	Male	435 (52.7)	222 (51.5)	213 (53.9)	0.483
	Female	391 (47.3)	209 (48.5)	182 (46.1)	
Age (year)	< 18	62 (7.5)	31 (7.2)	31 (7.8)	0.077
	18–45	202 (24.5)	97 (22.5)	105 (26.6)	
	46–64	388 (46.9)	221 (51.3)	167 (42.3)	
	≥65	174 (21.1)	82 (19.0)	92 (23.3)	
Marriage	Married	650 (78.7)	346 (80.3)	304 (77.0)	0.245
	Others^b^	176 (21.3)	85 (19.7)	91 (23.0)	
Household size (Mean, SD)		1.53 (0.50)	3.60 (1.69)	4.06 (2.48)	< 0.01
PCHI (CNY) (Mean, median)		9307 (6250)	10,555 (7178)	7951 (5280)	< 0.001^a^
Hospital level	county or less level	211 (25.5)	136 (31.6)	75 (19.0)	< 0.001
	municipal or higher level	615 (74.5)	295 (68.4)	320 (81.0)	
Inpatient times (Mean, SD)		4.59 (6.73)	5.71 (8.80)	3.36 (2.76)	< 0.001
Inpatient days (Mean, SD)		49.03 (66.21)	65.51 (80.06)	35.42 (42.76)	< 0.001
Inpatient expenses (Mean, median)		70,897 (56159)	74,284 (63095)	67,150 (50828)	< 0.001^a^
NCMS reimbursement (CNY^c^) (Mean, median)		34,742 (27593)	33,515 (28228)	36,081 (26889)	0.926^a^
CII reimbursement (CNY) (Mean, median)		7023 (3581)	8045 (4582)	5908 (3005)	< 0.001^a^
OOP (CNY) (Mean, median)		28,499 (22316)	32,531 (25050)	24,100 (18225)	< 0.001^a^
NDMC (CNY) (Mean, median)		4433 (2860)	4480 (2985)	4384 (2784)	0.743^a^
Indirect cost (CNY) (Mean, median)		9244 (2000)	10,248 (2000)	8148 (1500)	0.106^a^
Financial assistance from kin	Yes	233 (28.2)	146 (33.9)	87 (22.0)	< 0.001
	No	593 (71.8)	285 (66.1)	308 (78.0)	
Medical assistance	Yes	198 (24.0)	140 (32.5)	58 (14.7)	< 0.001
	No	628 (76.0)	291 (67.5)	337 (85.3)	
Medical debt					
Incidence	Yes	568 (68.8)	283 (65.7)	285 (72.2)	< 0.05
	No	258 (31.2)	148 (34.3)	110 (27.8)	
Median load (CNY) (Mean, median)		36,768 (20000)	39,847 (20000)	33,407 (20000)	0.845a

^a^the rank sum test

^b^others contains unmarried, divorced and widowed

^c^exchange rate 650 CNY = US$100 at the end of 2017

### Coping strategies for medical expenses

**Fig. 1:**
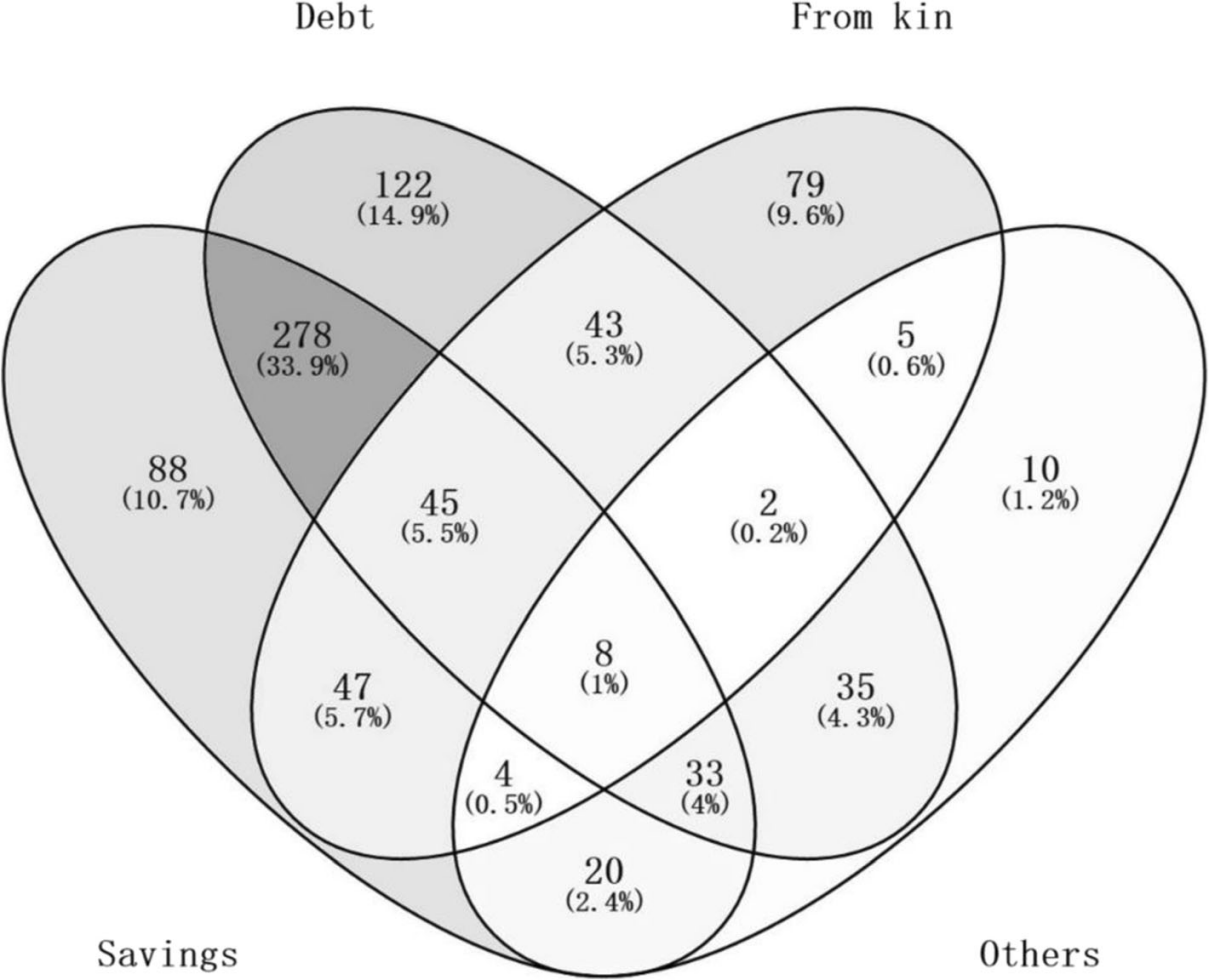
Coping strategies for medical expenses

**Fig. 2:**
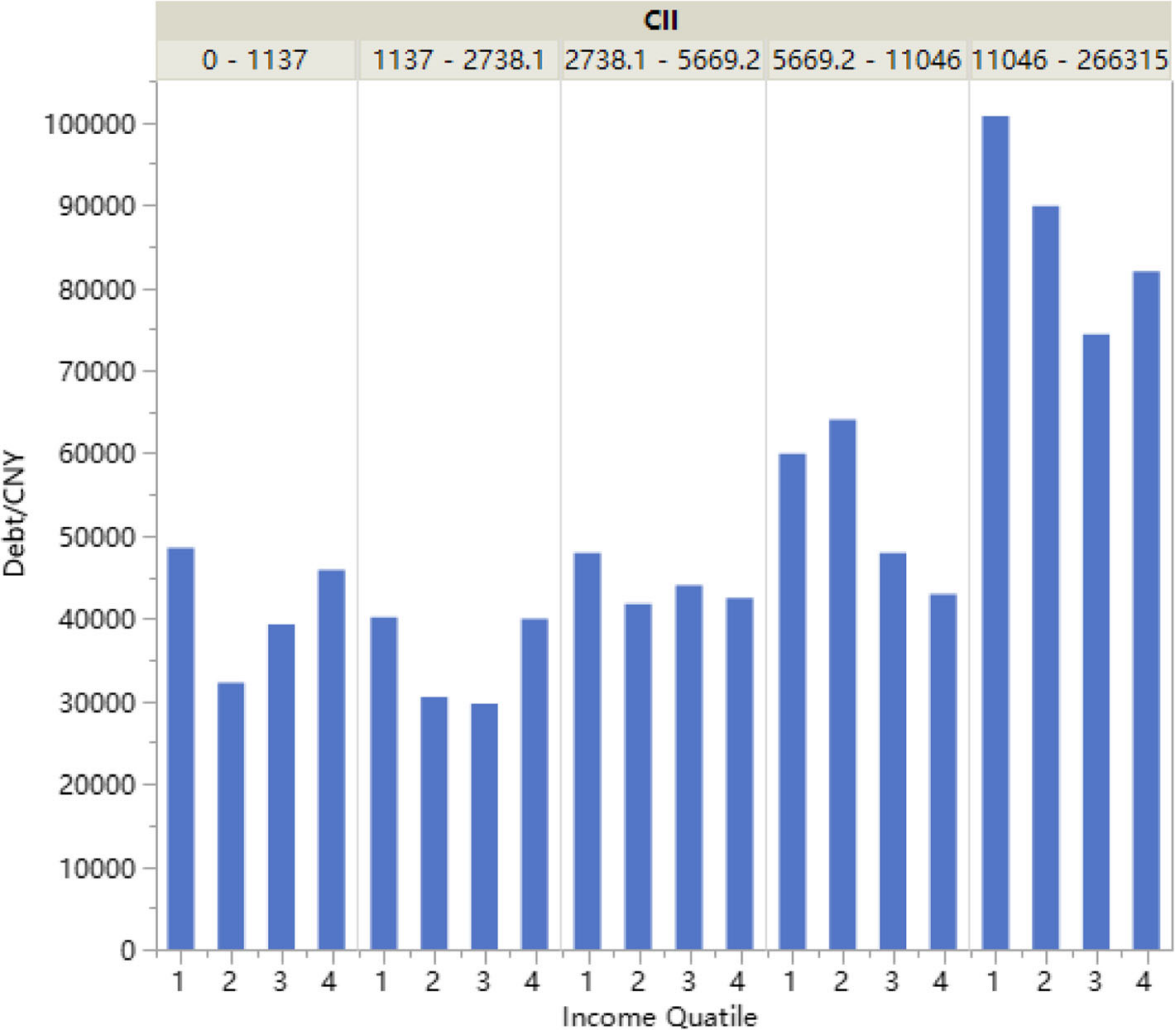
Average medical debt load in income and CII reimburesement groups

Among the 826 respondents, the most common coping strategy that households adopted upon incurring large health expenditure was debt (about 68.8%), followed by using savings (60.1%). In addition, 28.4% of patients received assistance from their children or relatives. As shown in Fig. 1, when the coping strategies for paying medical OOP expenses were combined, the highest combination was “debt and savings” (accounting for 33.9%), whereas 14.9% of respondents relied on debt alone to pay for their healthcare expenses, and only 10.7% of respondents were solely dependent on savings. Figure 2 shows that the patients with highest CII had the highest medical debt load. More household income indicated less medical debt.

### Determinants of medical debt

Table 3 presents the results of the two-part model. Column I displayed the estimated coefficients of the logit model, and column II presented their z-values. The estimates of the debt load equation coefficients and their t-values were indicated in columns III and IV, respectively. In the prevalence equation, financial assistance from kin decreased the incidence of medical debt, with a coefficient of − 1.468 (*P* < 0.001). The household size was > 4, and NCMS reimbursement ratio and NDMC were the risk factors of medical debt. For medical debt load, the patients hospitalized in municipal or high-level hospitals (coef = 0.187, *P* < 0.05) had higher CII reimbursement ratio (coef = 1.995, *P* < 0.05) and NDMC (coef = 0.121, *P* < 0.001), with increased debt amount. IE was a risk factor of medical debt load. The patients in quartiles 3 and 4 had higher medical debt, with coefficients of 0.340 (*P* < 0.01) and 0.569 (*P* < 0.001), respectively. PCHI was also a protective factor of debt load. In the random effect analysis of the two counties, likelihood-ratio test versus logistic/linear regression showed that regional-level differences were statistically significant during linear regression (Table 3).

**Table 3:** Estimated two-part models of rural patients’ medical debt

Independent variables		Prevalence(*n* = 826)		Log (debt load)(*n* = 568)	
		Coefficient (SE)	z-Stat.	Coefficient (SE)	z-Stat.
Age (years)	≤17	Ref		Ref	
	18–44	0.518 (0.388)	1.34	−0.046 (0.157)	−0.29
	45–64	0.657 (0.402)	1.63	−0.045 (0.167)	−0.27
	65+	−0.236 (0.413)	− 0.57	− 0.329 (0.181)	−1.81
Gender	Male	Ref		Ref	
	Female	0.060 (0.172)	0.35	−0.104 (0.067)	−1.55
Marriage	Married	Ref		Ref	
	Others	−0.263 (0.247)	−1.07	−0.074 (0.105)	− 0.71
Household size	≤3	Ref		Ref	
	≥4	0.422 (0.187)^a^	2.25	0.062 (0.072)	0.86
PCHI (least to highest)	Quartile 1	Ref		Ref	
	Quartile 2	0.230 (0.247)	0.93	−0.144 (0.094)	−1.53
	Quartile 3	−0.019 (0.257)	− 0.07	−0.306 (0.097)^b^	−3.16
	Quartile 4	−0.404 (0.255)	−1.59	−0.236 (0.099)^a^	−2.40
Hospital level	County or less	Ref		Ref	
	Municipal or higher	0.054 (0.232)	0.23	0.187 (0.096)^a^	1.96
Inpatient days		−0.001 (0.002)	−0.39	− 0.002 (0.001)^b^	− 3.43
Inpatient expenses (least to highest)	Quartile 1	Ref		Ref	
	Quartile 2	−0.178 (0.241)	−0.74	0.001 (0.097)	0.10
	Quartile 3	−0.010 (0.262)	−0.04	0.340 (0.100)^b^	3.40
	Quartile 4	−0.404 (0.308)	−0.69	0.569 (0.117)^c^	4.88
NCMS reimbursement ratio		1.633 (0.819)^a^	1.99	−0.120 (0.346)	−0.35
CII reimbursement ratio		2.574 (2.103)	1.22	1.995 (0.785)^a^	2.54
NDMC		0.225 (0.067)^b^	3.36	0.121 (0.029)^c^	4.12
Indirect cost		0.035 (0.020)	1.75	−0.006 (0.008)	− 0.77
Financial assistance from kin	No	Ref		Ref	
	Yes	−1.468 (0.206)^c^	−7.14	−0.189 (0.095)^a^	−1.98
Medical assistance	No	Ref		Ref	
	Yes	0.420 (0. 231)	1.81	0.078 (0.084)	0.93
Constant		−2.384 (1.074)^a^	−2.22	9.411 (0.479)^c^	19.67
Likelihood-ratio test vs. logistic/linear regression					
Chiba2		0.00		6.83^b^	

^a^Indicates significance at the 0.05 level

^b^Indicates significance at the 0.01 level

^c^Indicates significance at the 0.001 level

## Discussion

### Prevalence of medical debt in patients with CII in rural areas of China

Health coverage refers to equitable access to health services for all at an affordable cost and the extent to which health service costs are covered [46]. The rates of medical debt provide insights into the household’s economic burden and the level of financial protection. In this paper, the data suggested that medical debt was a very common and serious phenomenon among patients with critical illness living in rural areas of China. The incidence of medical debt was considerably higher among patients with critical illness than in other studies that focused on common patients. For example, Christy et al. found that the medical debt rate among low-income households in a mid-western state of the US was 43%. Collins et al. revealed that 24% of adults incurred medical debt in 2010 [24, 47]. These findings implied that medical debt is a serious indication of economic burden brought by disease, and patients with critical illness are poorly protected from financial risk.

The actual burden to patients with critical illness could be increased by the imperfect financial markets in rural China. Many studies that focused on the coping strategies that households employ to deal with health shocks found that borrowing is a key mechanism used by rural households to mitigate the effects of health expenses [40, 48]. However, because of the imperfect financial markets, many households in rural areas of developing countries lack access to formal credit; thus, much of the borrowing is informal (mostly from money lenders with relatively high interest, others being relatives and neighbors) in nature [49]. Leive et al. showed that whether households pay for their healthcare expenses through borrowing depends not only on the ability to provide a collateral for obtaining loan but also on the availability of social capital [19, 50]. In the present study, 28.2% of patients received assistance from their children or relatives, which could significantly reduce the incidence of medical debt. Studies showed that transferring health expenses to offspring or other family networks is an important means of financial support [19, 20]. However, depending on social support and lacking formal credit could lead to increased economic burden permanently. Under this condition, rural households facing difficulties to pay for healthcare expenses may even decide to forgo treatment because of poor access to formal debt and high cost of informal debt, thereby leading to severely negative effects due to possible disease complications in the long run [51]. Providing formal credit with zero or small interest, such as in Care Payment Program [52], to patients with critical illness may help reduce some of the negative consequences of medical debt.

### Factors of prevalence and medical debt load in rural areas of China

The common distribution of medical debt (including incidence and load) for patients with critical illness could be attributed to several factors. Financial assistance from kin was a key determinant of medical debt among patients with critical illness. Given the Chinese traditional views of mutual aid, these patients could rely more on family networks by transferring the health expenses to their offspring or other family networks. Sons or daughters provided high financial assistance to parents with critical illness due to the restriction of filial piety. With this alternative payment method, the occurrence of medical debt naturally decreased. In addition, household income was a protective factor of medical debt load. High-income households were more resilient to economic risks and capable of reducing medical debt load than low-income households. This result was in line with other studies [25, 43].

NDMC includes hospital accommodation, transportation, accommodation, and other expenses. It is a risk factor of debt incidence and medical debt load. With the continuous development of China’s economy, most rural working age people leave their areas to work in big cities [53]. XT and YQ are counties with a high proportion of migrant workers. However, the medical insurance territorial reimbursement policy requires these workers to go home when sick, thereby increasing their transportation and accommodation expenses. Moreover, the mountainous terrain of YQ causes local traffic inconvenience. If patients go to the city to seek inpatient services, the cost of transportation and accommodation is much higher than in other counties. Thus, the economic level of YQ is much lower than that of XT, but their NDMCs were similar. In the rural mountainous areas of China, limited transportation, poverty, and poor primary health services could worsen the medical services for diagnosis and treatment [54].

High IE was also a risk factor of medical debt load. When patients face increased medical expenditure, they borrow money to pay for these expenses. The average IE of patients with critical illness was more than 8640 USD. Under the pressure of increased medical expenses, having increased debt load is reasonable.

The inadequate increase in reimbursement may be one of the factors related to medical debt. CII reimbursement ratio was a risk factor of medical debt load. The CII deductibles were 12,000 CNY in XT and 8000 CNY in YQ. These standards were based on the local PCHI, but many poor families could not even pay for the threshold. Referring to the PCHI in Table 2, it was too high for patients to enjoy the subsidies. When CII only covers the part above the threshold, households must meet the other part of the costs of care themselves [55]. International studies suggested that public subsidies for health programs frequently benefit richer people more than poorer people [56–58]. High CII means patients with serious or urgent illnesses have increased medical expenses. Thus, they need to borrow more money to treat their critical illness.

Finally, more inpatients with critical illness seeking medical care at municipal or high-level hospitals may be another factor that increased medical debt load due to increased inpatient expenditures, such as supplementary drugs and additional tests. It also resulted in high costs of supplementary transportation and board and lodging. It may restrict reimbursement for inpatient care because it tends to encourage a more cautious and restrictive approach by the NCMS. Patients hospitalized at municipal or high-level hospitals may deal with higher deductibles and lower reimbursement rates than those hospitalized at county or low-level hospitals [59]. The complications of critical illness and its comprehensive treatment increased non-reimbursable expenses. The increase in the use of high-level hospitals is probably related to the complications of critical illness to some extent. In rural China, the healthcare delivery system is composed of village clinics, THCs, and hospitals at the county level [60]. The government should enhance the service capability of the counties’ three-level health institutions, provide training to improve doctors’ skills, and increase medical facilities. Patients could be hospitalized in county-level and township hospitals rather than large hospitals outside the county.

This study has several limitations that need to be recognized. First, the analysis was based on self-reported data. The information on the amount each household borrowed or the various costs related to health services was probably subjected to recall or other forms of self-reported bias. Second, the households that were too poor to seek healthcare were not captured in the analysis, thereby underestimating the economic risk of critical illness. Third, patients covered by CII may have higher medical expenditures and medical debt than those not covered by CII; thus, the study focused on the former. Future research could include patients covered by NCMS and compare these two populations. Finally, the findings of this study were from a cross-sectional survey data. Longitudinal population-based studies about the effects of medical debt on subsequent health-seeking behavior are needed to understand the magnitude and extent of financial hardship resulting from medical debt and how they increase future vulnerability to shocks.

## Conclusion

Affordability is still one of the biggest problems in healthcare systems worldwide, especially in developing countries. This paper chose medical debt as an indicator to indirectly reflect the long-term burden of patients with critical illness. This paper also provides important information about the distribution of medical debt and its influencing factors among patients with critical illness in rural areas of China. The results showed that the prevalence of medical debt in rural patients with critical illness was high.

NDMC was a key factor of medical debt. Patients with critical illness who incurred high NDMCs in rural areas were more likely to pay for their medical OOP expenses by borrowing money. Suitable transportation and accommodation subsidies could also be provided to patients with critical illness, such as those with cancer or undergoing dialysis. Although reimbursement from health insurance continues to be improved, it is still inadequate to reduce the burden on patients with critical illness due to the sharp increase in health expenses and high deductible. Thus, introducing formal risk-pooling mechanism of critical illness and increasing its reimbursement in poor areas are crucial and beneficial.

## Supplementary information

**Additional file 1.** Questionnaire for Patients with Critical Illness

## Data Availability

The datasets used and/or analyzed in this study are available from the corresponding author on reasonable request.

## Abbreviations

OOP: Out-of-Pocket
CII: Critical illness insurance
UHC: Universal Health Coverage
CMS: Cooperative Medical System
NCMS: New Cooperative Medical Scheme
URBMI: Urban Resident-based Basic Medical Insurance
THCs: Township Health Centers
XT: Xiantao
YQ: Yuqing
CNY: China Yuan
GDP: Gross Domestic Product
PPS: Probability Proportionate to Size
PCHI: Per capita household income
IE: Inpatient expense
ERR: Effective Reimbursement Rate
NDMC: Non-direct Medical Cost
