# Supplementary material for: What contributes to medical debt? Evidence from patients in rural China
Source: BMC Health Serv Res. 2020 Jul 28;20:696. doi: 10.1186/s12913-020-05551-5 (PMC7388505; doi:10.1186/s12913-020-05551-5)
Supplement: Supplementary file 1 — Additional file 1. Questionnaire for Patients with Critical Illness [file 12913_2020_5551_MOESM1_ESM.docx]

**No.：□□□□□□□ Questionnaire for Patients with Critical Illness**

**Family address：＿＿＿＿province＿＿＿＿＿city＿＿＿＿＿＿＿county**

Hello! I am an investigator at Huazhong University of Science and Technology. I want to know about your consultation services, costs and burdens after illness, and to provide a basis for improving national policies for critical illness insurance. Participation in this study is completely voluntary. You agree to this survey, and we will be grateful. Thank you! This survey will take about 20-30 minutes.

| **One. Patient characteristics** | | | | | | |
| --- | --- | --- | --- | --- | --- | --- |
| 1. **Patient name** | | | | | |  |
| 1. **Medical certificate number** | | | | | |  |
| 1. **Gender: 1. male 2. female** | | | | | | ___ |
| 1. **Patient age (years)** | | | | | | ___ ___ |
| 1. **Interviewees: 1. The patient himself 2. Family members 3. Others _________** | | | | | | ___ |
| 1. **Your family population is _________, school-age children are _________** | | | | | |  |
| 1. **What is your current marital status? 1. Married 2. Unmarried 3. Divorced 4. Widowed** | | | | | | ___ |
| 1. **Are you currently living alone? 1. yes 2. no** | | | | | | ___ |
| 1. **What is your highest level of education? 1. Never attended school 2. Elementary school 3. Junior high school or equivalent 4. High school or equivalent 5. Technical secondary school 6. Junior college 7. Undergraduate and above** | | | | | | ___ |
| 1. **What do you do now? 1. Wage work or self-employed 2. Farming 3. Unemployed (unemployed, unemployed) 4. Retired 5. Disability**   **6. School students / pre-schoolers 7. Others (please specify)** | | | | | | ___ |
| **Two. Inpatient and outpatient services and expenses** | | | | | | |
| 1. **How long have you been ill so far?** | | | | | | ___ |
| 1. **How many times were you hospitalized in 2016? _________ times for _________ days** | | | | | | ___ |
| **13.** **Cost of each hospitalization** | | | | | | |
| **Medical expenses during hospitalization** | **1st** | **2nd** | **3rd** | **4th** | **5th** | **others（>5）** |
| 13.1 name of hospital |  |  |  |  |  |  |
| 13.2 days in hospital |  |  |  |  |  |  |
| 13.3 Transportation expenses |  |  |  |  |  |  |
| 13.4 Room and board |  |  |  |  |  |  |
| 13.5 Companionship |  |  |  |  |  |  |
| 13.6 Self-purchased drug / materials |  |  |  |  |  |  |
| **14. Since your illness, have you ever been diagnosed by a doctor and you have not been hospitalized? 1. Yes 2. No (skip to Q17)** | | | | | | ___ |
| **15.** **How many times? _________ times** | | | | | | ___ |
| **16. If happed, what are the main reasons?**  **1. Economic difficulties 2. unnecessary 3. Self-inflammation is hopeless 4. No time 5. Long distance 6. Other ____ (please specify)** | | | | | | ___ |
| **17.** **The last discharge was due to: 1. doctor's request (skip to Q19) 2. own request 3. other reasons (skip 19)** | | | | | | ___ |
| **18.** **If you ask to be discharged yourself, the main reasons are: 1. Bad effect 2. self-considered illness 3. economic reasons 4. other** | | | | | | ___ |
| **19.** **How many times did you go to the clinic for the critical illness in 2016? _________ times** | | | | | | ___ |
| **20.** **Cost of each clinic** | | | | | | |
| **Medical expenses during clinic** | **1st** | **2nd** | **3rd** | **4th** | **5th** | **others（>5）** |
| 20.1 Outpatient time |  |  |  |  |  |  |
| 20.2 Name of Outpatient hospital |  |  |  |  |  |  |
| 20.3 Total Outpatient Cost |  |  |  |  |  |  |
| 20.4 out-of-pocket Outpatient Cost |  |  |  |  |  |  |
| 1. **Since your illness, have you ever needed an outpatient review and did not go? 1. Yes 2. No (skip to Q24)** | | | | | | ___ |
| 1. **How many times? _________ times** | | | | | | ___ |
| 1. **If happed, what are the main reasons?**   **1. Economic difficulties 2. unnecessary 3. Self-inflammation is hopeless 4. No time 5. Long distance 6. Other ____ (please specify)** | | | | | | ___ |
| 1. **Does the patient have an outpatient chronic illness card: 1. Yes 2. No** | | | | | | ___ |
| **Three. Family economic situation and other** | | | | | | |
| 1. **n 2016, the total annual household income: _______ yuan** | | | | | |  |
| 1. 26.1 In 2016, expenditures on family education, hydropower, etc .: _______ yuan | | | | | |  |
| 26.2 Household food expenditure _______ yuan | | | | | |  |
| 26.3.1 Expenses for inpatient and outpatients other than critical illness | | | | | |  |
| 26.3.2 Inpatient and outpatient medical expenses of other family members | | | | | |  |
| 26.2.3 Home-purchased drugs (such as those for other chronic diseases) _________ yuan | | | | | |  |
| 1. **How much was your family lost to work in 2016? ______yuan** | | | | | | ___ |
| 1. **Is your home listed as a local subsistence guarantee household: 1. Yes 2. No** | | | | | | ___ |
| 1. **Are you a registered cardholder for targeted poverty alleviation: 1. Yes 2. No** | | | | | | ___ |
| 1. **The cost of your treatment comes from (multiple choices): 1. Family savings 2. Property for sale 3. Loans 4. Donations 5. From kin _______ yuan 6. Others (please specify) _______** | | | | | | ___ |
| 1. **How much is the family borrowing for treating? (You only need to ask when you choose 3 from Q30) _______yuan** | | | | | | ___ |
| 1. **What do you think of the financial burden of the cost of medical treatment on your family?** 2. **1. No burden at all 2. Light burden 3. Fair 4. Heavy burden 5. Heavy burden** | | | | | | ___ |
| 1. **Do you think the current medical insurance has reduced your financial burden?**   **1. Significant reduction 2. Decreased, but not obvious 3. Not at all** | | | | | |  |

*Your phone (mobile): ____________*

*Do you have any good suggestions for the NRCMS policy? ____________________________________________________________________________________________________________________________________*

**Thank you for your cooperation! wish you healthy!**

**Ask the interviewer to self-examine and sign on the spot Interviewer Sign：**______________ **Date** _____________
